# Eliminating medicine waste in a Finnish university hospital — a qualitative study

**DOI:** 10.1186/s40545-019-0188-8

**Published:** 2019-10-02

**Authors:** Teijo Peltoniemi, Reima Suomi

**Affiliations:** Information Systems Science, Department of Management and Entrepreneurship, Turku School of Economics, University of Turku, Turku, Finland

**Keywords:** Medicine waste, Medicine supply chain, Hospital pharmacy, Information systems, Information technology

## Abstract

**Background:**

Medicine waste in hospitals leads to severe economic loss. This waste emerges for a number of reasons. Medicines are often ordered in too large quantities, which leads to stock expiring without being dispensed. Wastage can also be a consequence of poor management practices. Technical aids, such as automatic dispensers, have been suggested to reduce waste, but they too have shortcomings. Information systems can arguably contribute to waste reduction, but this area has not been widely researched.

In this exploratory case study, we scrutinized the management of medicines waste in a hospital from an information systems perspective and examined how information systems are used to manage the medicine supply chain and medicine waste. Our research case was a Finnish university hospital, its central pharmacy, and, more widely, the medicine supply chain within the hospital.

**Methods:**

This is a qualitative case study, based on data gathered through interviews and a survey and a review of other information sources, including annual reports and other relevant collateral. The study participants included pharmacy staff members and other hospital staff involved in medicine supply. The interviews were conducted in two rounds, first capturing the main themes and then exploring them further in the later study stages.

**Results:**

The findings outline a picture of unfit technology and inconsistent and unreliable information. This is compensated for by manual practices and processes that cause an excessive administrative burden and ultimately increased wastage. An infrequent ordering process combined with the lack of recycling practices increase the wastage even more.

**Conclusion:**

Medicine supply and waste management remain a manual administrative task. Inconsistent information and unfit information systems make this task challenging, and the process relies on the medicine supply staff’s experience and assumptions.

## Introduction

Medicine waste leads to significant economic losses at the societal level. For example, the United Kingdom (UK) National Health Service estimates the annual losses caused by prescription medicine waste at £300 million [1]. The problem is not specific to the UK; the World Health Organization (WHO) suggests that most health systems are struggling with inefficiencies in the medicine supply chain [2]. In hospital surroundings, the amount can be high too: Toerper et al. [3] suggest that in busy wards with high patient and medicines turnover, the wastage can be as high as 29% of medicine expenses.

Pharmaceutical or medicine waste refers to the medicine-related disposals generated in the course of healthcare activities. It comprises expired, unused, and contaminated medicines. It is one type of healthcare waste [4]. Other types are radioactive and genotoxic waste, sharps, i.e., syringes and needles, and pathological waste. WHO’s main concerns with healthcare waste relate to the health and environmental risks [4]. Such waste can lead to air pollution and drinking water contamination, which threaten health. According to WHO, 15% of healthcare waste is hazardous, i.e., toxic or infectious [4]. Typically, the risks are exposed through inadequate processing practices, such as inadequate incineration or disposing of untreated waste in landfill. In addition to the usual waste, medicines can be lost during the logistic process or stolen in a criminal act [5].

Medicine waste is problematic, as it is difficult to measure. In Finland, for example, the total losses are estimated as “high”, but the exact amount is neither monitored nor reported by healthcare providers or authorities [6]. In a report based on information from various Finnish hospital districts, the losses from wasted medicines were estimated at 6 million euros in 2015 [7]. One way to measure medicines waste is to compare the acquisition costs to the actual consumption. However, this requires detailed medicines tracking, which is currently not standard practice in Finnish hospital districts. In addition, medicines disposed of in wards might not be reported and hence would not be treated as waste, as described below. Medicine waste has been studied in different accounts [8, 9].

Losses often occur due to poor management practices [10]. Technical solutions have been introduced to overcome these. One example is the automatic medicine dispenser, which maintains an accurate and up-to-date inventory. An automatic dispenser, which keeps track of different medicines’ expiry dates, enables more efficient rotation of medicines, which can release considerable annual savings [11]. The shortcomings are that it is not able to predict future demand and hospital-wide deployment is an extensive project.

Improving the hospital medicine supply process by increasing delivery frequency has been suggested to reduce waste [3], as in the manufacturing sector, in which just-in-time processing is a standard way to operate. Simulation tools can optimize the medicine delivery process [12], and they have been used successfully for the dispensing process [13].

Information systems are arguably an integral part of any hospital’s operation, and they therefore play a role in medicine waste management. However, research in this area is scarce. To fill the research gap, we studied the management of the medicine supply chain and medicine waste in a Finnish university hospital from an information systems perspective. Our aim was to examine how medicine supply and medicine waste are managed in hospital surroundings, how information systems support this process, and what the main shortcomings are.

## Methods

### Introducing the case

Our exploratory case study is based on Turku University Hospital (hereafter TYKS) in the city of Turku. TYKS is the central hospital in the Hospital District of Southwest Finland (hereafter VSSHP), serving 28 municipalities in Western Finland. The hospital comprises 156 outpatient clinics and wards [14] and has a central pharmacy providing services to wards and clinics. The hospital pharmacy is part of Pharmaceutical Services of Southwest Finland, the pharmaceutical organization operating within the hospital district. The central pharmacy is headed by a lead pharmacist, and it employs various teams of pharmacists and staff working with medicines [15]. The pharmacists who work in wards are referred to as field pharmacists in this article. Not all wards are allocated a pharmacist; in those wards nurses deal with medicine delivery.

Medicine waste comprises expired, unused, and contaminated medicines. In addition, if a medicine package is opened but not fully consumed by the patient, it is considered waste. Notably, the bulk of medicine waste is usable medicines that are neither consumed nor recycled. The wastage in VSSHP was 858,371 euros in 2015 [7], and the overall medicine expenses were 54,096,132 euros. Wastage has remained between 1 and 2% of total medicine acquisition expenses between 2007 and 2015, according to data from a hospital pharmacy representative (K. Torniainen, email communication, Feb 6, 2017).

Certain Finnish healthcare service providers, including university hospitals, must hold a mandatory reserve stock of medicines [16]. Hospitals therefore acquire more medicines than they will use to maintain the required stock levels—this is another source of waste. For example, in 2015, the value of expiring emergency stock in VSSHP was 95,700 euros (K. Torniainen, email communication, Feb 6, 2017).

### Research method

This exploratory case study uses qualitative methods and data from different sources. Qualitative methods are not exceptional within the pharmacy domain [17, 18], which is hardly surprising given the patient-centric and humanist approach required in pharmaceutical care. From an information systems standpoint, a common failure is neglecting to fully understand the linkage between the technology and the organization using it—focusing only on technology and ignoring people and organizations often leads to a failed implementation or poor adoption of an information system [19–21].

The first phase of the research was familiarization with TYKS’ medicine supply and pharmaceutical services. We interviewed three central pharmacy staff (interviewees A–C) and one field pharmacist (interviewee D) asking open questions. This allowed us to conceptualize the research context and outline themes for further investigation. The interviewees A–C, as key central pharmacy staff, were allocated by the hospital. This was required to set the scene. The hospital allocated interviewee D based on the hospital unit, a ward with relatively high medicine turnover and typical practices for inventory keeping.

In the next phase we conducted interviews with a field pharmacist (interviewee E) and a nurse dealing with medicines in a ward (interviewee F), deep diving into some of the themes identified in the first phase. The themes included: a) how medicines demand and loss are managed, b) the practicalities of associated work processes, and c) the unofficial, ‘shadowy’ side of this work. The interviewees were again selected based on the unit they work in. To receive an adequate view, and to prevent bias, a busy ward with a high medicine turnover and a quieter ward with a low medicine turnover were selected.

Finally, we conducted an online survey to gather further data about the research context and themes. Survey invitations were sent to the field pharmacist and chief nurse mailing lists. Recipients were asked to forward the invitation to all staff managing medicines in their respective units. The survey was anonymous, and the invitation may have been forwarded to others also. Thirty individuals responded, including five field pharmacists, 19 nurses accountable for medicine handling in a ward and six unspecified roles. The questions were voluntary, and therefore the number of respondents varied question by question. The respondents are described in Table 1.

**Table 1:** Research participants

Participant type	Interview	Survey	Initials used in data analysis
Pharmacist – central pharmacy	3		Interviewees A, B, and C
Field pharmacist	2	5	Interviewees D and E
Other medical supply staff in wards	1	19	Interviewee F
Other staff		6	

The survey included four background questions to establish the recipient’s role within the medicine supply process, followed by eight open-ended, voluntary questions concentrating on views and problems the respondents perceived in relation to information technology (IT) supporting their work across the medicine supply process. The final section consisted of 14 questions relating to the recipient’s attitudes towards medicine wastage and recycling, in which the respondents were asked to respond to claims such as ‘medicine loss is a severe problem’ on a scale between 1 and 5, whereby 1 was ‘fully disagree’ and 5 was ‘fully agree’.

The background documentation reviewed included VSSHP annual reports [22–24], induction material for new employees [15], and a summary of financials relating to medicine waste in VSSHP (K. Torniainen, email communication, Feb 6, 2017). The authors and two MSc students participated in the first visit at the hospital as well as the survey setup.

## Results

### Uncertain operating environment

Medicine delivery is an information-intensive area, and the roles and tasks are managerial and administrative. However, there often seem to be issues with the integrity and reliability of information. Whereas TYKS has an automated central pharmacy that maintains an accurate inventory of centrally stocked medicines, wards rely on a manual inventory (interviewees D, E, and F). There are also a number of other manual tasks included with a ward’s daily medicine supply operations. For example, nurses compile information from various systems, such as the patient record system, to estimate the daily demand (interviewee D).

Ordering of medicines from the central pharmacy is carried out with an order entry IT application. This application neither provides information about the central pharmacy stock levels nor the expiry dates of the available medicines. Ensuring the medicines will have adequate expiry dates requires using another application, and given the hectic work rhythm in the ward, there is scarcely time to do this. This may result in ordering a large quantity of medicines that expire shortly. Often, orders are based on rules of thumb rather than facts (interviewee D).

One of our findings was that medicines disposed in wards are not monitored. In the hospital this is called ‘mandatory loss’ (interviewee D). Only medicines returned to the central pharmacy are incorporated in the waste figures. It is impossible to measure total waste, as only unopened and unexpired medicine packages can be returned to the pharmacy. Although VSSHP’s annual medicine loss is higher than in other hospital districts [6], it is possible that VSSHP monitors the wastage more accurately than other hospital districts.

The central pharmacy is responsible for placing orders to medicine wholesalers by compiling ward orders into one centralized order (interviewee B). The pharmaceutical staff are reluctant to perform this task, as the IT systems involved are considered unintuitive and deemed ‘buggy’ (interviewee C).

Furthermore, the expiry date information in the medicine wholesalers’ IT systems was described as unreliable (interviewees B and C). There are automatic controls in the central pharmacy’s inventory that trigger predefined order templates when stock levels fall below predetermined limits, but these are perceived as poorly defined and hence unusable (interviewee C).

To facilitate medicine recycling between hospital units, field pharmacists have introduced an unofficial email group (interviewee D). Recycling and using this email group is voluntary rather than mandatory. Waste reduction is not an explicit and monitored goal for staff.

When respondents were asked whether they thought the management of the hospital monitores medicine loss, 18 respondents gave a neutral answer. When asked whether medicine loss was monitored in wards, more respondents were inclined to agree than disagree. Twenty-three respondents agreed that medicine loss burdens the hospital’s economy. In interviews, none of the interviewees were able to specify any loss figures. Despite this, 23 agreed that their intention was to recycle medicines whenever possible.

### Technological aids for sense making

Twelve different IT systems and external data sources involved in medicine ordering were identified in the survey. Based on these findings, we conclude that complete information is challenging to piece together. Furthermore, various manual paper-based lists and notes are utilized in wards. For example, an interviewee explained that they use manually maintained spreadsheets to track incoming medicines (interviewee E). Four survey respondents pointed out that expiry dates should be visible in the order entry application rather than being accessible only through another IT application.

In the survey, we asked respondents to specify the main benefits they receive from IT systems. Ten respondents mentioned process-related topics. These respondents perceived that IT systems speed up and boost work processes. A further nine respondents mentioned benefits relating to accurateness of information; generally, respondents perceived that they received useful, accurate and up-to-date information from IT systems they use.

Controversially, when asked about the main shortcomings of IT, 12 respondents mentioned usability-related issues. Generally, according to respondents, IT systems are old-fashioned, unintuitive, and generally poor to use. Two respondents mentioned the lack of integration within the dispersed IT system landscape causing information gaps between hospital units and duplicate work when inputting the same data into different systems. Five respondents mentioned the quality of information being poor and incomplete. For instance, the expiry dates for medicines are not available and inventories are not up-to-date. According to the annual reports, incidents with the IT infrastructure have been a recurring theme, having jeopardized the patient safety in multiple occasions [23, 24].

When drilling down to the order process, the respondents perceived having limited tools for forecasting medicine demand. In addition, the practices seemed to vary between wards. It was not unusual to rely on experience rather than factual data (six respondents). Only three respondents claimed to base the order on facts, such as expiry dates and costs.

Conversely, when we asked respondents’ attitudes towards how well IT systems support them in forecasting medicine demand, 21 respondents were neutral or had a positive response. Similarly, 28 respondents were either neutral or positive when gauging their capability to estimate short-term medicine demand. The figure was the same when asking about the capability to estimate demand in the long run.

### The process and the organization

It is common sense that busier units, such as emergency wards, order more frequently than hospital units with less medicine consumption. There are also other factors which affect the ordering frequency. One field pharmacist explained that medicines are ordered a maximum of three times a week and the goal is two deliveries a week (interviewee E).

One interviewee mentioned that the introduction of field pharmacists had had a significant impact on decreasing medicine loss (interviewee A). According to another interviewee, it had been said that a field pharmacist earns their own salary through the savings made by recycling expiring medicines (interviewee E).

The survey contained an open-ended question in which we asked respondents to specify how medicine loss could be improved and whether they had any further comments regarding medicine demand management. Only one comment was about technology: one respondent pointed out that there should be an automated inventory in the ward. Most of the comments were process- and organization-related.

Seven respondents commented that the staff ordering medicines in wards simply should not order too large quantities. This was backed up by our interview findings, whereby interviewees confessed to ordering large quantities just in case or to save time (interviewee D). Inconsistent expiry date information seemed a repeating theme throughout the survey data,. Issues with other incomplete data, such as information about patient turnover, were scarcely mentioned.

Seven comments in this section were about central pharmacy and how they could improve the service. Again, these comments were about receiving better information about expiry dates. Another issue was too large package sizes: often, specific medicines are ordered for one patient and unconsumed medicines in the package become mandatory loss.

Six respondents mentioned people-related topics. For example, respondents believed that experienced, dedicated staff would decrease medicine loss. One respondent mentioned that medicine loss began to decrease when they nominated a single person to be accountable for medicine orders and returns. One respondent believed that if they were allocated a field pharmacist they would more likely receive the ‘right amount’ of medicines delivered to the ward.

### Shadow recycling practices

There are four main elements in the medicine supply organization: the central pharmacy, the ward, the wider group of staff handling medicines, and the field pharmacists. Field pharmacists seemed to be a tight-knit team, forming a bond between the wards and the central pharmacy. Other staff handling medicines seemed to be unfamiliar with the official and unofficial practices involved with medicine supply.

One of the shadowy practices seemed to be the unofficial recycling email group, which was used to facilitate recycling of medicines between wards. One interviewee explained that it is used to advertise medicines expiring in the ward to facilitate recycling (interviewee E). This interviewee believed that only field pharmacists belong to the ring; however, 14 survey respondents claimed to belong to the ring, although only five of them were field pharmacists. Interviewee D believed that medicines are recycled only between units that have a dedicated field pharmacist. Interviewee F, who worked in a medicine handling role in a ward, but was not a pharmacist, had never heard of the recycling ring.

Interviewee E noted that the discussion is very active in the recycling email group. The interviewee explained that they are constantly discussing and innovating different ways to reduce medicine waste (interviewee E). There also seemed to be some peer pressure involved: when survey respondents were asked to estimate whether they recycle because their peers do, more respondents agreed than disagreed.

In terms of field pharmacists, the annual reports show that the team has grown over the years: in 2014–2015 there were 7–8 pharmacists working in wards (within the whole hospital district) [22, 23], whereas in 2016 this number had grown to 17 [24]. Unfortunately, we do not have loss figures for 2016 onwards available.

### Summary of finding

We have summarized the main findings in Table 2.

**Table 2:** Main findings from information systems standpoint

Finding	Description
Inconsistent expiry information	The medicine inventory in wards is maintained manually and there is no accurate information on expiry dates. The expiry information on the wholesaler’s ordering system can be incorrect.
Lack of integration	As the IT systems are not integrated, staff need to manually crosscheck information from various applications when estimating medicines demand and placing orders. Staff often avoid this manual task and base their estimations on rules of thumb.
Lack of official recycling scheme	Recycling is not a managed process, and it is based on voluntary action and an unofficial email ring.
Poor usability of IT applications	Applications appear unintuitive and onerous to use, which leads to further avoidance of their use.
Infrequent ordering process	As the ordering process is a rather heavy and manual operation, it is more economical to order infrequently and in larger quantities than needed.
Inaccurate metrics	Accurate figures on medicine waste are unavailable, given that only medicines returned to the pharmacy are counted as waste. This leads to more confusion in terms of managing and reducing waste.

## Discussion

The inconsistent information and poor usability of IT systems leads to compensating with rules of thumb and manual processes. An example of this is the ordering process, whereby the ordered quantity is often based on assumptions or the previous order. The snowball effect accumulates when the central pharmacy combines a number of arbitrary orders from wards into a centralized order to a medicine wholesaler. Sometimes the inventory information provided by a wholesaler is incorrect, leading to ordering a large quantity of medicines expiring shortly. This all results in unavoidable medicine waste.

Furthermore, accurate figures on waste are not available. This is because only medicines returned to the pharmacy are taken into account. The lack of metrics makes it hard to monitor the wastage.

The medicine supply within the hospital is an infrequent process, and medicines are ordered in larger quantities than needed. This is understandable, as ordering is a time-consuming process, and there is a cost associated with the delivery. Medicines are often available only in unnecessarily large packages, and unconsumed medicines cannot be returned.

The ward staff accept these shortcomings and seek improvements through amending their personal work practices. For example, the respondents suggested that voluntary personal actions should be considered to improve recycling, whilst the unfit ordering process was not questioned.

The staff seem to take full accountability in reducing medicine loss and use ‘shadow’ channels to accomplish this since official practices are not available. There is no official communication on waste reduction or related goals.

The field pharmacist role is pivotal, and these pharmacists form a key group within the organization, linking different parties together and driving improvements. However, the shortcomings with information and IT lead to manual processes and increased administrative overheads, which are hard to overcome.

A study limitation is that the participants did not include many interfacing groups, such as the senior management of the hospital district or IT systems vendors. This shortcoming could be overcome by expanding the scope of the study to cover these stakeholders in future research. Furthermore, the exploration could be expanded to other hospital districts to gauge a broader view on the issue of medicine waste.

## Conclusions

The medicine supply process is characterized by many shortcomings in terms of IT systems usability and availability of consistent information. For example, information on stock levels and expiry dates in wards is maintained manually, and the information is rarely accurate. This leads to excessive administrative work, which is avoided by using assumptions rather than facts in decision making. The ordering process, however, is infrequent and emphasizes large quantities. This, combined with the absence of an efficient recycling process for medicines between hospital units, culminates in expiring stocks, which inevitably generate large quantities of medicine waste. The waste is not monitored accurately, and there is uncertainty about the actual quantity of medicine waste. The medicine delivery staff is, however, committed to reduce waste and seeks to mitigate it through unofficial channels, such as informal email groups.

Based on this study, three actions could be considered to gain quick wins in terms of reducing the waste. Firstly, an application to facilitate medicine recycling between units could be considered. Secondly, informal communications could be facilitated through digital channels to help build the wider community of medicine supply staff. This would contribute to sharing information and best practice on medicine recycling. Thirdly, the intra-hospital medicine ordering process should be more frequent, whereby smaller batches would be supplied more frequently.

Three more far-reaching and sustainable solutions are also recommended. Firstly, the information architecture should be reconsidered to ensure the integrity of data and reduce the manual processing. Secondly, automatic inventory management in wards could help to reduce waste caused by expiring medicines. Finally, waste should be monitored and reported more accurately, as otherwise it is challenging to plan actions and objectives for reducing the waste. However, this may require a wider cultural as well as technical change. These call for further research, which could take the form of design science and action research, for example.

## Data Availability

Please contact the author for data requests.

## Abbreviations

IT: Information Technology
TYKS: Turku University Hospital
UK: The United Kingdom
VSSHP: The Hospital District of Southwest Finland
WHO: World Health Organization
